# Occurrence of Multi-Drug-Resistant *Escherichia coli* in Chickens, Humans, Rodents and Household Soil in Karatu, Northern Tanzania

**DOI:** 10.3390/antibiotics10091137

**Published:** 2021-09-21

**Authors:** Valery S. Sonola, Abdul S. Katakweba, Gerald Misinzo, Mecky I. N. Matee

**Affiliations:** 1 Africa Centre of Excellence for Innovative Rodent Pest Management and Biosensor Technology Development (ACE IRPM & BTD), Sokoine University of Agriculture, P.O. Box 3110, Morogoro 67125, Tanzania; katakweba@sua.ac.tz; 2 Department of Wildlife Management, College of Forestry, Wildlife and Tourism, P.O. Box 3073, Morogoro 67125, Tanzania; 3 Livestock Training Agency (LITA), Buhuri Campus, P.O. Box 1483, Tanga 21206, Tanzania; 4 Institute of Pest Management, Sokoine University of Agriculture, P.O. Box 3110, Morogoro 67125, Tanzania; 5 Department of Veterinary Microbiology, Parasitology and Biotechnology, College of Veterinary Medicine and Biomedical Sciences, Sokoine University of Agriculture, P.O. Box 3019, Morogoro 67125, Tanzania; gerald.misinzo@sacids.org; 6 Department of Microbiology and Immunology, School of Medicine, Muhimbili University of Health and Allied Sciences, P.O. Box 65001, Dar es Salaam 11103, Tanzania; mateemecky@yahoo.com

**Keywords:** *Escherichia coli*, antibiotic resistance, humans, rodents, chickens, soil, isolates

## Abstract

We investigated antibiotic resistance profiles of *Escherichia coli* among 960 samples obtained from chickens (236), humans (243), rodents (101) and soil (290). *E. coli* was isolated from 650 (67.7%) samples. Isolation frequency varied significantly between chickens, humans, rodents and soil samples, being 81.6%, 86.5%, 79.2% and 31.0%, respectively (*p* < 0.001). Resistance rates were particularly higher against imipenem (79.8%), cefotaxime (79.7%) and tetracycline (73.7%) and moderate against amoxicillin-clavulanate (49.4%). Overall, 78.8% of the isolates were multidrug-resistant (MDR) among which, 38.8%, 25.1%, 12.9% and 2.5% exhibited resistance to three, four, five and six different classes of antibiotics, respectively. Multidrug-resistant *E. coli* were observed in 27.7%, 30.3%, 10.8% and 10.0% of the isolates from chickens, humans, rodents and soil samples, respectively. Our results show high levels of antimicrobial resistance including MDR in *E. coli* isolated from chickens, humans, rodents and soil samples in Karatu, Northern Tanzania. Comprehensive interventions using a one-health approach are needed and should include improving (i) awareness of the community on judicious use of antimicrobial agents in humans and animals, (ii) house conditions and waste management and (iii) rodent control measures.

## 1. Introduction

Antibiotic resistance is currently a serious problem worldwide that threatens human, animal and environmental health [1]. If the situation remains unmanaged by 2050, higher human mortalities, severe economic losses and a significant drop in livestock production are expected [2]. *Escherichia coli* is the major cause of urinary tract infections and neonatal meningitis in humans [3], and it also causes avian colibacillosis, a serious infectious disease in poultry [4]. Other conditions caused by *E. coli* in chickens include yolk sac infections, pericarditis, peritonitis and osteomyelitis [5]. *E. coli* is a commensal microbe in humans and chickens that carries and spreads resistance genes to other pathogens [6], threatening public health. Rodents that invade human habitats carry and transmit different zoonotic pathogens including MDR *E. coli,* threatening human health [7,8]. The interaction between rodents, humans, livestock and their environment in households can facilitate sharing of antibiotic-resistant bacteria and their resistance genes [9]. Studies have documented that, in rural areas where poultry farming is common, household soils are contaminated with higher antibiotic residues from humans and animals [10,11,12,13], leading to an increase in and spread of antibiotic resistance genes, involving contamination of the environment [14]. Previous studies have pointed out the potential role of peridomestic rodents in the spread of resistant bacteria to humans and domestic animals, either directly or through the environment [7,14,15]. Thus, the interaction between humans, livestock and peridomestic rodents is of public health concern, since it has the potential to cause infections that are difficult to treat [9]. In Karatu district in Northern Tanzania, studies have shown intense interactions between rodents, humans and animals, leading to infectious disease epidemics [16,17,18,19,20]. However, none of these studies screened bacteria for antimicrobial resistance. We hypothesize these interactions can cause the transmission of resistant bacteria, fueling the spread of antimicrobial resistance (AMR) in the community. We conducted this study in Karatu district in the northern zone of Tanzania to isolate and phenotypically screen for antimicrobial resistant *E. coli* among humans, chickens, rodents and the soil in households that keep indigenous chickens. This cross-sectional study determined phenotypic AMR patterns of *E. coli* isolated from rodents, humans, chickens, and their environment in households in an area where their interaction is intense.

## 2. Results

### 2.1. Isolation of Escherichia coli from the Samples

*E. coli* was isolated from 650 (67.7%) samples (Table 1). Isolation of *E. coli* varied significantly between chickens, humans, rodents and soil samples at 81.9%, 86.5%, 80.2% and 31.0%, respectively (*p* < 0.001).

### 2.2. Antibiotic Resistance of E. coli Isolates from Chickens, Humans, Rodents and Soil

**Figure 1:**
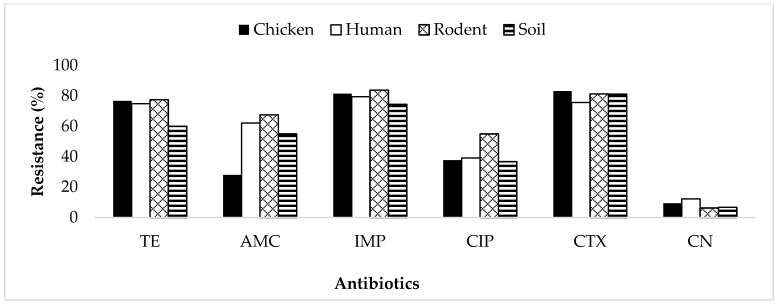
Resistance rates of *E. coli* isolates to different classes of antibiotics. TE = tetracycline; AMC = amoxicillin-clavulanate; IMP = imipenem; CIP = ciprofloxacin; CTX = cefotaxime; CN = gentamycin.

Overall, the *E. coli* isolates were resistant to tetracycline (73.7%), amoxicillin-clavulanate (49.4%), imipenem (79.8%), ciprofloxacin (40.2%), cefotaxime (79.7%) and gentamycin (9.7%), as shown in Figure 1. The overall resistance rates were 34.5%, 38.6%, 13.7% and 13.1% for isolates from chickens, humans, rodents and soil, respectively. Increased resistance of isolates to imipenem, cefotaxime and tetracycline was observed in all types of samples (Figure 1).

### 2.3. Multidrug-Resistance (MDR) of Escherichia coli Isolates from All Samples

A total of 512 out of 650 isolates (78.8%), were resistant to three and above different classes of antibiotics. Most of the isolates (38.3%) were resistant to three classes of antibiotics, and 16 isolates (2.5%) were resistant to all classes where most of them were from chicken (seven isolates) and human (five isolates) samples (Table 2). We observed significant variation in the occurrence of MDR isolates from different types of samples being higher in humans (30.3%) and chickens (27.7%) compared to rodents (10.8%) and soils (10.0%) (*p* < 0.001).

### 2.4. Prevalence of MDR Isolates of E. coli in Different Locations of Karatu

**Figure 2:**
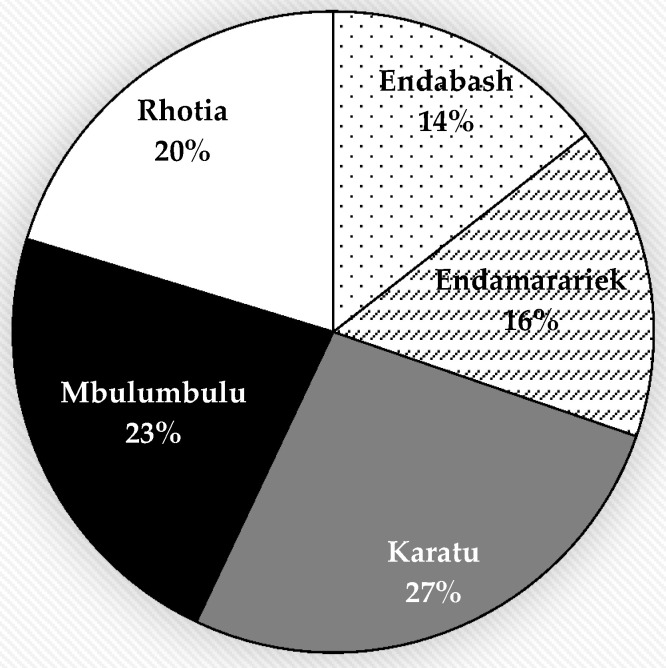
Distribution of MDR *E. coli* isolates in different areas of Karatu district.

Among 512 MDR isolates, 74, 81, 153, 137 and 116 were isolated from the samples collected in Endabash, Endamarariek, Karatu, Mbulumbulu and Rhotia wards, respectively (Figure 2).

All MDR *E. coli* isolates from chickens, humans, rodents and soil samples were distributed in the wards as shown in Table 3. Significant variation in the prevalence of MDR isolates was observed between the wards, being higher in samples from Karatu (21.1%) compared to those from Endabash (11.4%) ward (*p* < 0.001) (Table 3).

**Figure 3:**
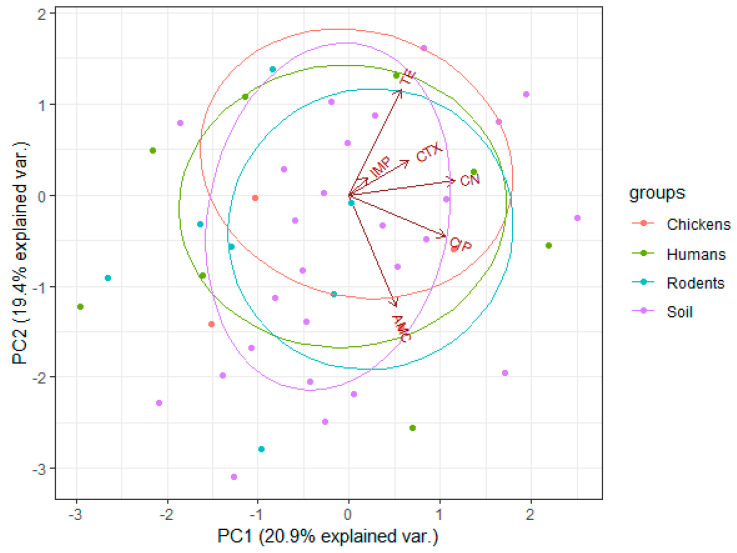
Principal component analysis (PCA) biplots of the antibiotic resistance profiles of *E. coli* isolated from chickens, humans, rodents and soil samples. For principal component 1 (PC1), the *X*-axis expresses susceptibility of the isolates to the drugs, while for principal component 2 (PC2), the *Y*-axis expresses resistance of the isolates. Arrows indicate the antibiotics that were used during resistance screening. The dots represent the *E. coli* isolates that were resistant to the tested antibiotics with respect to their sample sources. The ellipses indicate a 95% confidence interval of the respective isolates from the same sample source.

As shown in Figure 3, along principal component 1 (PC1), the arrow for gentamycin (CN) is very close to 0 (*X*-axis), followed by that of ciprofloxacin (CIP), indicating their respective lower variances compared to other drugs, indicating that isolates were more susceptible to CN followed by CIP. The arrow for amoxicillin–clavulanate (AMC) is far (high deviation) from the PC1 axis (Susceptibility), indicating relatively higher resistance rates of isolates to AMC compared to CN and CIP. Along the principal component 2 (PC2), the arrows for tetracycline (TE), imipenem (IMP), cefotaxime (CTX) and AMC are close to PC2 (*Y*-axis), showing higher variances compared to other drugs, which implies higher resistance rates of isolates to these drugs. The large positive loadings for TE, IMP and CTX indicate a greater and positive correlation between them in terms of resistance patterns. The overlapping of ellipses indicates that the proportions of resistant isolates did not vary significantly across sample sources.

### 2.5. Phenotypic Patterns of MDR E. coli Isolates from Chickens, Humans, Rodents and Soil

MDR *E. coli* isolates from chickens displayed different resistance patterns where TE-IMP-CTX (7.4%), TE-IMP-CIP-CTX (3.5%) and TE-AMC-CIP-CTX (3.1%) were the most common as shown in Table 4.

MDR isolates from human samples displayed TE-IMP-CTX (4.5%), TE-AMC-IMP-CTX (4.9%) and TE-AMC-IMP-CIP-CTX (5.1%) as the common patterns of resistance (Table 5).

Among 70 MDR *E. coli* isolates from rodent samples, 8 isolates (4.5%), 9 isolates (1.4%) and 22 isolates (3.4%) displayed TE-IMP-CTX, TE-AMC-IMP-CTX and TE-AMC-IMP-CIP-CTX as common patterns of resistance, respectively (Table 6).

For MDR isolates from soil samples, TE-IMP-CTX (2.0%) and TE-AMC-IMP-CTX (1.8%) were the common resistance patterns, as shown in Table 7 below. Overall, the combination TE-IMP-CTX was the most common pattern appearing in isolates from all types of samples.

## 3. Discussion

We conducted this study in Karatu because of high and frequent interactions among rodents, humans and chickens that have been reported in previous studies and shown to cause the spread of infections [16,17,18,19,20]. To the best of our knowledge, this is the first study in Tanzania to simultaneously screen MDR *E. coli* in chickens, humans, rodents and soil. In total, 650 out of 960 samples (67.7%) were positive for *E. coli.* We found high resistance of isolates against imipenem (79.8%), cefotaxime (79.7%), tetracycline (73.7%) and amoxicillin-clavulanate (49.4%) compared to ciprofloxacin (40.2%) and gentamicin (9.7%). Indeed, the principal component 1 (PC1) indicated that isolates were more susceptible to gentamycin followed by ciprofloxacin, and relatively higher resistance rates of isolates to amoxicillin-clavulanate. The principal component 2 (PC2), indicated that tetracycline, imipenem, cefotaxime and amoxicillin-clavulanate were close to PC2 (*Y*-axis) showing higher variances compared to other drugs, which implies higher resistance rates of isolates to these drugs. Interestingly, large positive loadings for tetracycline, imipenem and cefotaxime indicated greater and positive correlation between them in terms of resistance patterns. The overlapping of ellipses indicates that the proportions of resistant isolates did not vary significantly across sample sources, indicating widespread presence of AMR *E.coli*. Most of the highly resisted antibiotics are readily available and can be obtained over the counter without prescription [21,22,23].

We observed that 78.8% of all isolates were MDR *E. coli*, and that chicken (27.7%) and human (30.3%) isolates had significantly higher MDR isolates as compared to those recovered from rodents (10.8%) and soil (10.0%). Higher occurrence rates of MDR isolates in chickens and humans can be influenced by the frequent use and misuse of antibiotics in humans and poultry in Karatu [21,22,24,25]. The presence of MDR *E. coli* isolates in rodents indicates their potential role as hosts or vectors that can spread MDR *E. coli* to humans and chickens in Karatu and corresponds with other studies in Kenya, Germany, Canada and Vietnam [14,26,27,28] that associated rodents with carriage and spread of MDR and virulent *E. coli* strains in communities. The isolation frequency of MDR *E. coli* from soil was 10.0%, which is close to the 12.6% reported in Bangladesh [29]. This indicates that household soils are potential reservoirs of MDR *E. coli,* possibly due to poor disposal of sewages and poultry manure in households. Previous studies in Tanzania have revealed that mismanagement of human and livestock wastes in households contaminates the soil with *E. coli* [10,30,31]. In our study, MDR isolates exhibited different resistance patterns. However, the combination of tetracycline, imipenem and cefotaxime (TE-IMP-CTX) was the most common in all types of samples, highlighting their frequent use in humans and poultry. The increased use of cefotaxime and tetracycline during disease treatment and prevention in poultry has been widely documented worldwide, resulting in widespread antibiotic resistance [32,33,34,35]. Interestingly, we found a higher isolation frequency of MDR *E. coli* in samples from Karatu (21.1%), which is an urban area and district headquarter compared to Endabash ward (11.4%), a rural area. Concomitant with this, we found a higher level of interaction between humans, chickens, rodents and their environment in urban households, coupled with a higher level of antimicrobial use and low levels of waste management, which may have facilitated the spread of MDR *E. coli* [9,36]. Finally, we acknowledge that our findings provide preliminary insight on the magnitude and pattern of AMR *E. coli* in humans, chickens, rodents and their environment and not transmission dynamics. Molecular studies of AMR genes of isolates from humans, chickens, rodents and soil will be required to determine cross-transmission of superbugs among different hosts and the environment in the area. Nonetheless, these findings should alert public health officials to take the necessary interventions, including raising public awareness, on the appropriate use of antimicrobial agents and proper hygiene measures, including waste disposal.

## 4. Materials and Methods

### 4.1. Study Location

The study was conducted in Karatu district in the Northern zone of Tanzania between June 2020 and March 2021. Karatu is located between latitudes 3°10′ and 4°00′ S and longitude 34°47′ E. The district has a population of 230,166 people comprised of 117,769 men and 112,397 women with an average of 5 people per household [37]. Karatu has an altitude range of 1000 to 1900 m above sea level with two wet seasons annually (short rains between October and December and long rains from March to June).

### 4.2. Sampling Strategy

The study population included all households keeping local chickens, while the sampling frame was the list of these households. Five wards (Karatu, Endabash, Endamarariek, Mbulumbulu and Rhotia) were purposively selected based on population density (at least 16,000), number of households with chickens, and household size of at least 5 people. Households were randomly selected from a list provided by a livestock field officer at ward level by using a table of random numbers. At the household level, house owners’ permission was used in trapping the rodents where areas for trapping in the surrounding environments relied on signs of rodents’ activities. For each household, one adult (18 years and above) resident and one mature (7 months) scavenging chicken participated in microbiological sampling (1 fecal and 1 nasal swab). Additionally, at least one rodent (in-house rat, peridomestic rat or both) could be captured, as well as one soil sample collected per household. The selection of adult humans and mature chickens was based on the assumption that old individuals have been exposed to the interaction with rodents for a long time than young ones and hence are more likely to facilitate sharing of infections.

### 4.3. Trapping of Rodents for Sample Collection

Live trapping of 101 rodents was carried out using Modified-Sherman traps baited with peanut butter. Each captured rodent was euthanized by using di-ethyl-ether. Deep pharyngeal swabs and intestines were aseptically collected from the carcasses.

### 4.4. Collection of Samples from Humans, Chickens and Soil

A total of 960 samples was obtained from chickens (236), humans (243), rodents (101) and soil (290). Cloacal swabs were collected from randomly picked scavenging local chickens and human stools in households. Additionally, soil samples were randomly collected from five points in the household yards and mixed to compose 1 pooled soil sample [10]. Thereafter, all samples were stored in sterile containers at –4 °C and transported using Cary Blair transport medium to Tanzania Veterinary Laboratory Agency (TVLA) laboratory in Arusha for bacteriological analyses.

### 4.5. Culture, Isolation and Identification of E. coli Isolates

The specimens were plated onto MacConkey agar (Oxoid ltd., Detroid, MI, USA) and incubated aerobically at 37 °C for 24 h. Presumptive colonies of *E. coli* were subjected to a combination of four biochemical tests—indole, methyl red, Voges–Proskauer and citrate utilization (IMViC)—as well as motility tests for identification as per Quinn et al. [38]. *E. coli* strain American Type Culture Collection (ATCC) 29,522 was used as a standard organism.

### 4.6. Antibiotic Susceptibility Testing of E. coli Isolates

An antibiotic susceptibility test was performed by using Kirby–Bauer disc diffusion method on Mueller–Hinton Agar plates (Oxoid, Basingstoke, UK) with commercially available discs as described by [39]. The antibiotics tested were tetracycline (30 μg), imipenem (10 μg), chloramphenicol (30 μg), gentamicin (10 μg), ciprofloxacin (5 μg), cefotaxime (30 μg) and amoxicillin-clavulanate (20 μg/10 μg). Pure colonies of the identified lactose fermenters were emulsified into 5 mL of sterile saline. The suspensions were adjusted to achieve turbidity equivalent to 0.5 McFarland standard solutions, emulsified using sterile cotton swabs onto Mueller–Hinton Agar plate, and incubated at 37 °C for 16 to 18 h. After incubation the inhibition zone of each antimicrobial agent was measured, and results were interpreted according to the CLSI standards [39]. *E. coli* strain American Type Culture Collection (ATCC) 29,522 was used as standard organism. An isolate was considered to be multidrug-resistant if it was non-susceptible to three or more drugs from different classes of antibiotics [40].

### 4.7. Statistical Analyses

Isolation frequencies of *E. coli* and antibiotic resistance profiles of isolates were entered into Microsoft Excel version 2010 (Microsoft Corporation, Redmond, WA, USA) and their percentages calculated by descriptive statistics. Association between categorical variables was analyzed using Chi-square (Fisher’s exact and Pearson’s) test. Principal component analysis (PCA) was used to describe the distribution of *E. coli* resistant isolates with respect to their sample sources and antibiotic resistance profiles. PCA was performed using R Statistical Package Windows version 3.4.2. Statistical significance was accepted at *p <* 0.05.

## 5. Conclusions

The level of antimicrobial resistance, including multi-drug-resistant *E. coli* seen in isolates from humans, chickens, rodents and soil raises the possibility of widespread transmission of resistance genes and bacteria in the studied area, with the possibility of causing infections that are difficult to treat. The antibiotics used in this study are the ones that are commonly used in the area for treating both human and animal infections, implying that they have limited success in their intended use. Comprehensive interventions, using a one-health approach, would be required to control the situation. Such measures should include improving (i) awareness of the community on judicious use of antimicrobial agents in humans and animals, (ii) house conditions and waste management and (iii) rodent control measures.

## Figures and Tables

**Table 1:** Isolation frequencies of *Escherichia coli* from different samples.

Type of Samples	Total Number of Samples n (%)	Positive Samples n (%)	Chi-Square	*p*-Value
Chickens	288 (30.0)	236 (81.9)	X^2^ = 147.58, df = 3	<0.001
Humans	281 (29.3)	243 (86.5)		
Rodents	101 (10.5)	81 (80.2)		
Soil	290 (30.2)	90 (31.0)		
Total	960 (100.0)	650 (67.7)		

**Table 2:** Antibiotic resistance and MDR rates of *Escherichia coli* isolates from chickens, humans, rodents and soil samples.

Types of Sample Sources	Number of Antibiotic Classes to Which the Isolates Were Resistant, n (%)							Total Number of Isolates	MDR Isolates (3–6 Classes)	Chi-Square	*p*-Value
	0	1	2	3	4	5	6				
Overall n (%)	5 (0.8)	24 (3.7)	109 (16.8)	249 (38.3)	163 (25.1)	84 (12.9)	16 (2.5)	650 (100.0)	512 (78.8)		
Chickens	0 (0.0)	10 (1.5)	46 (7.1)	103 (15.8)	55 (8.5)	15 (2.3)	7 (1.1)	236 (36.3)	180 (27.7)	129.75 df = 3	<0.001
Humans	5 (0.8)	8 (1.2)	33 (5.1)	88 (13.5)	63 (9.7)	41 (6.3)	5 (0.8)	243 (37.4)	197 (30.3)	75.47, df = 3	<0.001
Rodents	0 (0.0)	2 (0.3)	9 (1.4)	25 (3.8)	20 (3.1)	22 (3.4)	3 (0.5)	81 (12.5)	70 (10.8)	16.16, df = 3	0.001
Soil	0 (0.0)	4 (0.6)	21 (3.2)	33 (5.1)	25 (3.8)	6 (0.9)	1 (0.2)	90 (13.8)	65 (10.0)	42.75, df = 3	<0.001

**Table 3:** Prevalence of MDR *E. coli* isolates from all types of samples in different wards in Karatu district.

Ward	Types of Sample Sources, n (%)				MDR Isolates	Chi-Square	*p*-Value
	Chickens	Humans	Rodents	Soil			
Overall n (%)	180 (27.7)	197 (30.3)	70 (10.8)	65 (10.0)	512 (78.8)		
Endabash	20 (3.1)	22 (3.4)	17 (2.6)	15 (2.3)	74 (11.4)	1.6571, df = 3	0.647
Endamarariek	32 (4.9)	27 (4.2)	10 (1.5)	12 (1.8)	81 (12.5)	18.532, df = 3	<0.001
Karatu	40 (6.2)	61 (9.4)	24 (3.7)	12 (1.8)	137 (21.1)	49.118, df = 3	<0.001
Mbulumbulu	50 (7.7)	42 (6.5)	8 (1.8)	16 (2.5)	116 (17.8)	43.571, df = 3	<0.001
Rhotia	38 (5.8)	45 (6.9)	11 (1.7)	10 (1.5)	104 (16.0)	39.44, df = 3	<0.001
Chi-Square	24.82, df = 4	25.03, df = 4	13.077, df = 4	2.00, df = 4			
*p*-value	<0.001	<0.001	0.011	0.736			

**Table 4:** Resistance patterns of 180 MDR *E. coli* isolates from chicken samples.

Chicken Samples	Number of Isolates (n)	Occurrence (%)	Antibiotic Resistance Patterns	Number of Antibiotic Classes
(n = 180)	10	1.5	AMC, IMP, CTX	3
	9	1.4	IMP, CIP, CTX	
	1	0.2	AMC, IMP, CIP	
	2	0.3	AMC, CIP, CTX	
	3	0.5	TE, AMC, CTX	
	2	0.3	TE, AMC, IMP	
	4	0.6	TE, CIP, CTX	
	48	7.4	TE, IMP, CTX	
	1	0.2	TE, IMP, CIP	
	2	0.3	TE, AMC, CTX	
	12	1.8	TE, CIP, CTX	
	2	0.3	TE, IMP, CIP	
	3	0.5	TE, CIP, CTX	
	2	0.3	TE, AMC, IMP	
	1	0.2	TE, IMP, CN	
	1	0.2	TE, AMC, CIP	
	1	0.2	TE, CIP, CTX, CN	4
	2	0.3	AMC, IMP, CTX, CN	
	4	0.6	AMC, IMP, CIP, CTX,	
	23	3.5	TE, IMP, CIP, CTX	
	1	0.2	TE, IMP, CIP, CN	
	20	3.1	TE, AMC, CIP, CTX	
	2	0.3	TE, AMC, IMP, CTX	
	2	0.3	TE, IMP, CTX, CN	
	10	1.5	TE, AMC, IMP, CIP, CTX	5
	3	0.5	TE, IMP, CIP, CTX, CN	
	1	0.2	TE, AMC, IMP, CTX, CN	
	1	0.2	TE, IMP, CIP, CTX, CN	
	7	1.1	TE, AMC, IMP, CIP, CTX, CN	6
Total	180	27.7		

AMC = amoxicillin-clavulanate; TE = tetracycline; IMP = imipenem; CTX = cefotaxime; CIP = ciprofloxacin; CN = gentamycin.

**Table 5:** Resistance patterns of 197 MDR *E. coli* isolates from human samples.

Human Samples	Number of Isolates (n)	Occurrence (%)	Antibiotic Resistance Patterns	Number of Antibiotic Classes
(n = 197)	17	2.6	AMC, IMP, CTX	3
	5	0.8	AMC, IMP, CIP	
	2	0.3	AMC, CIP, CTX	
	29	4.5	TE, IMP, CTX	
	10	1.5	TE, AMC, CTX	
	1	0.2	IMP, CIP, CTX	
	8	1.2	TE, AMC, IMP	
	7	1.1	TE, IMP, CIP	
	2	0.3	TE, AMC, CIP	
	2	0.3	TE, CIP, CTX	
	2	0.3	TE, CTX, CN	
	1	0.2	TE, IMP, CTX	
	1	0.2	TE, CIP, CTX	
	1	0.2	TE, IMP, CN	
	6	0.9	TE, AMC, IMP, CIP	4
	1	0.2	IMP, CIP, CTX, CN	
	1	0.2	TE, AMC, IMP, CN	
	1	0.2	TE, AMC, CTX, CN	
	1	0.2	AMC, IMP, CTX, CN	
	1	0.2	AMC, IMP, CIP, CN	
	32	4.9	TE, AMC, IMP, CTX	
	4	0.6	TE, AMC, CIP, CTX	
	9	1.4	TE, IMP, CIP, CTX	
	7	1.1	AMC, IMP, CIP, CTX	
	33	5.1	TE, AMC, IMP, CIP, CTX	5
	1	0.2	AMC, IMP, CIP, CTX, CN	
	4	0.6	TE, AMC, IMP, CTX, CN	
	2	0.3	TE, IMP, CIP, CTX, CN	
	1	0.2	TE, AMC, CIP, CTX, CN	
	5	0.8	TE, AMC, IMP, CIP, CTX, CN	6
Total	197	30.3		

AMC = amoxicillin-clavulanate; TE = tetracycline; IMP = imipenem; CTX = cefotaxime; CIP = ciprofloxacin; CN = gentamycin.

**Table 6:** Resistance patterns of 70 MDR *E. coli* isolates from rodent samples.

Rodent Samples	Number of Isolates (n)	Occurrence (%)	Antibiotic Resistance Patterns	Number of Antibiotic Classes
(n = 70)	3	0.5	AMC, IMP, CIP	3
	4	0.6	AMC, CIP, CTX	
	1	0.2	AMC, IMP, CTX	
	1	0.2	IMP, CIP, CTX	
	8	1.2	TE, IMP, CTX	
	1	0.2	TE, AMC, IMP	
	1	0.2	TE, CIP, CTX	
	1	0.2	TE, AMC, CTX	
	1	0.2	TE, AMC, IMP	
	3	0.5	TE, IMP, CIP	
	2	0.3	AMC, IMP, CIP, CTX	4
	9	1.4	TE, AMC, IMP, CTX	
	1	0.2	TE, AMC, CIP, CN	
	3	0.5	TE, IMP, CIP, CTX	
	5	0.8	TE, AMC, IMP CIP	
	22	3.4	TE, AMC, IMP, CIP, CTX	5
	1	0.2	TE, AMC, IMP, CIP, CN	
	3	0.5	TE, AMC, IMP, CIP, CTX, CN	6
Total	70	10.8		

AMC = amoxicillin-clavulanate; TE = tetracycline; IMP = imipenem; CTX = cefotaxime; CIP = ciprofloxacin; CN = gentamycin.

**Table 7:** Resistance patterns of 65 MDR *E. coli* isolates from soil samples.

Source of Samples	Number of Isolates (n)	Occurrence (%)	Antibiotic Resistance Patterns	Number of Antibiotic Classes
(n = 512)	1	0.2	AMC, IMP, CIP	3
	7	1.1	AMC, IMP, CTX	
	3	0.5	AMC, IMP, CTX	
	1	0.2	IMP, CIP, CTX, CN	
	1	0.2	IMP, CTX, CN	
	3	0.5	TE, AMC, CTX	
	1	0.2	TE, AMC, CIP	
	1	0.2	TE, CIP, CTX	
	2	0.3	TE, IMP, CIP	
	13	2.0	TE, IMP, CTX	
	12	1.8	TE, AMC, IMP, CTX	4
	4	0.6	TE, IMP, CIP, CTX	
	4	0.6	TE, AMC, CIP, CTX	
	4	0.6	AMC, IMP, CIP, CTX	
	1	0.2	TE, IMP, CTX, CN	
	1	0.2	AMC, IMP, CIP, CTX, CN	5
	1	0.2	TE, IMP, CIP, CTX, CN	
	4	0.6	TE, AMC, IMP, CIP, CN	
	1	0.2	TE, AMC, IMP, CIP, CTX, CN	6
Total	65	10.0		

AMC = amoxicillin-clavulanate; TE = tetracycline; IMP = imipenem; CTX = cefotaxime; CIP = ciprofloxacin; CN = gentamycin.

## Data Availability

The data presented in this study are available on request from the corresponding author. The data are not publicly available due to privacy restrictions.
